# Supplementary material for: Menopause and the Risk of Developing Age-Related Macular Degeneration in Korean Women
Source: J Clin Med. 2022 Mar 29;11(7):1899. doi: 10.3390/jcm11071899 (PMC8999594; doi:10.3390/jcm11071899)
Supplement: Supplementary file 1 [file jcm-11-01899-s001.zip › Supplemental_Table S1.pdf]

Supplemental Table S1. Characteristics of participants according to whether or not menopausal status was present before matching

	Pre-menopause	Menopause	Total	P-value
Nunber of women	97,651	33,598	131,249	
Median age (years)	47 [43–52]	54 [51–56]	49 [44–54]	<0.001
Age at inclusion (years)				<0.001
40–44	34,962 (35.8)	1,087 (3.2)	36,049 (27.5)	
45–49	25,984 (26.6)	4,675 (13.9)	30,659 (23.4)	
50–54	21,705 (22.2)	13,452 (40)	35,157 (26.8)	
55–59	15,000 (15.4)	14,384 (42.8)	29,384 (22.4)	
Year on inclusion				<0.001
2011	21,004 (21.5)	7,058 (21)	28,062 (21.4)	
2012	22,133 (22.7)	8,040 (23.9)	30,173 (23)	
2013	26,423 (27.1)	9,384 (27.9)	35,807 (27.3)	
2014	28,091 (28.8)	9,116 (27.1)	37,207 (28.3)	
SES				<0.001
Mid~high SES	94,322 (96.6)	32,659 (97.2)	126,981 (96.7)	
Low SES	3,329 (3.4)	939 (2.8)	4,268 (3.3)	
Region				<0.001
Urban area	50,726 (51.9)	19,124 (56.9)	69,850 (53.2)	
Rural area	46,925 (48.1)	14,474 (43.1)	61,399 (46.8)	
CCI				<0.001
0	72,180 (73.9)	21,112 (62.8)	93,292 (71.1)	
1	14,743 (15.1)	6,523 (19.4)	21,266 (16.2)	
≥2	10,728 (11)	5,963 (17.7)	16,691 (12.7)	
Parity in cohort				<0.001
0	93,311 (95.6)	33,512 (99.7)	126,823 (96.6)	
1	2,767 (2.8)	60 (0.2)	2,827 (2.2)	
≥2	1,573 (1.6)	26 (0.1)	1,599 (1.2)	
Cardiovascular disease before inclusion				<0.001
Absent	95,255 (97.5)	32,100 (95.5)	127,355 (97)	
Present	2,396 (2.5)	1,498 (4.5)	3,894 (3)	
Hypertension before inclusion				<0.001
Absent	81,145 (83.1)	24,489 (72.9)	105,634 (80.5)	
Present	16,506 (16.9)	9,109 (27.1)	25,615 (19.5)	
DM before inclusion				<0.001
Absent	89,391 (91.5)	28,796 (85.7)	118,187 (90)	
Present	8,260 (8.5)	4,802 (14.3)	13,062 (10)	
Dyslipidemia before inclusion				<0.001
Absent	76,231 (78.1)	19,638 (58.4)	95,869 (73)	
Present	21,420 (21.9)	13,960 (41.6)	35,380 (27)	
First antithrombotic agent before inclusion				<0.001
Absent	94,477 (96.7)	31,335 (93.3)	125,812 (95.9)	
Present	3,174 (3.3)	2,263 (6.7)	5,437 (4.1)	

DM, diabetes mellitus; CCI, Charlson comorbidity index; MHT, menopausal hormone therapy; SES, socioeconomic status
Data are expressed as number (%) or median (25th percentile, 75th percentile)
