# Supplementary material for: Menopause and the Risk of Developing Age-Related Macular Degeneration in Korean Women
Source: J Clin Med. 2022 Mar 29;11(7):1899. doi: 10.3390/jcm11071899 (PMC8999594; doi:10.3390/jcm11071899)
Supplement: Supplementary file 1 [file jcm-11-01899-s001.zip › Supplemental_Table S2.pdf]

Table S2. Odds ratios for risk of age-related macular degeneration according to menopause before matching Korea National Health Insurance Database, 2007-2020

	Unadjusted		Formula 1 [a]		Formula 2 [b]	
	OR (95% CI) [a]	P-value	OR (95% CI) [a]	P-value	OR (95% CI) [a]	P-value
Menopause						
Pre-menopause	1		1		1	
Menopause	2.054 (1.439–2.931)	<0.001	1.066 (0.738–1.54)	0.734	1.059 (0.731–1.532)	0.763
Age at inclusion (years)						
40–44			1		1	
45–49			5.966 (1.624–21.918)	0.007	5.95 (1.617–21.891)	0.007
50–54			16.38 (4.699–57.096)	<0.001	16.218 (4.636–56.728)	<0.001
55–59			29.183 (8.411–101.255)	<0.001	28.412 (8.123–99.378)	<0.001
SES						
Mid~high SES			1		1	
Low SES			0.782 (0.248–2.465)	0.675	0.741 (0.234–2.34)	0.609
Region						
Urban area			1		1	
Rural area			0.782 (0.248–2.465)	0.641	0.917 (0.643–1.308)	0.632
CCI						
0			1		1	
1			0.929 (0.581–1.485)	0.758	0.901 (0.563–1.444)	0.666
≥2			0.735 (0.429–1.258)	0.261	0.691 (0.4–1.194)	0.185
Parity in cohort						
0			1		1	
1			3.354 (0.411–27.35)	0.258	3.321 (0.406–27.141)	0.263
≥2			0 (0-)	0.98	0 (0-)	0.98
CVD before inclusion						
Absent					1	
Present					1.3779 (0.617–3.086)	0.434
Hypertension before inclusion						
Absent					1	
Present					0.854 (0.549–1.33)	0.486
DM before inclusion						
Absent					1	
Present					1.638 (1.027–2.614)	0.038
Dyslipidemia before inclusion						
Absent					1	
Present					0.966 (0.653–1.43)	0.863
First antithrombotic agent before inclusion						
Absent					1	
Present					1.057 (0.507–2.204)	0.883

CCI, Charlson comorbidity index; CI, confidence interval; CVD, cardiovascular disease; DM, diabetes mellitus; MHT, menopausal hormone therapy; OR, odds ratio; SES, socioeconomic status

a ORs were adjusted for age per 5 years, SES, regrion, CCI, parity

b ORs were adjusted for age per 5 years, SES, regrion, CCI, parity, CVD, hypertension, DM, dyslipidemia, use of antithrombotic agent
